# Early versus delayed anticoagulation in acute ischemic stroke with atrial fibrillation according to infarct volume and location: A prespecified subgroup analysis of the OPTIMAS randomized controlled trial

**DOI:** 10.1177/17474930261441297

**Published:** 2026-03-30

**Authors:** Philip S Nash, Jonathan G Best, James Lyon, James K Ruffle, Thanyalak Amornpojnimman, Rom Mendel, Chris Foulon, Hakim-Moulay Dehbi, Norin Ahmed, Liz Arram, Maryam Balogun, Kate Bennett, Ekaterina Bordea, Emilia Caverly, Marisa Chau, Hannah Cohen, Mairead Cullen, Caroline J Doré, Stefan T Engelter, Robert Fenner, Gary A Ford, Aneet Gill, Rachael Hunter, Martin James, Archana Jayanthi, Gregory YH Lip, Sue Massingham, Macey L Murray, Iwona Mazurczak, Amalia Ndoutoumou, Bo Norrving, Jenny Philip, Hannah Sims, Nikola Sprigg, Tishok Vanniyasingam, Parashkev Nachev, Nick Freemantle, David Doig, David J Werring, the OPTIMAS Investigators

**Affiliations:** 1 Stroke Research Centre, Department of Translational Neuroscience and Stroke, UCL Queen Square Institute of Neurology, London, UK; 2 High Dimensional Neurology Group, Department of Translational Neuroscience and Stroke, UCL Queen Square Institute of Neurology, London, UK; 3 Groupe d’Imagerie Neurofonctionnelle (GIN), Institut des Maladies Neurodegeneratives-UMR, CNRS, Bordeaux, France; 4 Comprehensive Clinical Trials Unit, Institute of Clinical Trials and Methodology, UCL, London, UK; 5 Department of Haematology, Cancer Institute, UCL, London, UK; 6 University College London Hospitals NHS Foundation Trust, London, UK; 7 Neurology and Neurorehabilitation, University Department of Geriatric Medicine, FELIX PLATTER, University of Basel, Basel, Switzerland; 8 Medical Sciences Division, University of Oxford, Oxford, UK; 9 Royal Devon & Exeter Hospital, and University of Exeter Medical School, Exeter, UK; 10 Liverpool Centre for Cardiovascular Science at University of Liverpool, Liverpool John Moores University and Liverpool Heart & Chest Hospital, Liverpool, UK; 11 Department of Clinical Medicine, Aalborg University, Aalborg, Denmark; 12 MRC Clinical Trials Unit, Institute of Clinical Trials and Methodology, UCL, London, UK; 13 Department of Clinical Sciences, Department of Neurology, Skåne University Hospital, Lund University, Lund, Sweden; 14 Stroke Trials Unit, Division of Mental Health and Clinical Neuroscience, Faculty of Medicine and Health Sciences, University of Nottingham, Nottingham, UK

**Keywords:** Atrial fibrillation, ischemic stroke, anticoagulation, timing, infarct volume, intracranial hemorrhage

## Abstract

**Background:**

Randomized trials have demonstrated that early anticoagulation after acute atrial fibrillation-associated ischemic stroke is safe and non-inferior to delayed initiation. Whether anticoagulation should be delayed in people with larger infarcts is uncertain.

**Aims:**

To investigate whether ischemic stroke infarct volume, measured precisely by segmentation, modifies the treatment effect of early anticoagulation with a direct oral anticoagulant (DOAC).

**Methods:**

We did a prespecified secondary analysis of OPTIMAS (NCT: 03759938), a randomized, parallel-group, open-label trial with blinded outcome assessment which randomized people with acute ischemic stroke and atrial fibrillation to early initiation of any licensed DOAC, within 4 days of onset, or delayed initiation 7–14 days from onset. The primary outcome was a composite of recurrent ischemic stroke, symptomatic intracranial hemorrhage (ICH), and systemic arterial embolism within 90 days. A central neuroimaging laboratory determined infarct volume using diffusion-weighted magnetic resonance imaging (MRI) using a validated deep learning segmentation model; on computed tomography (CT), infarcts were segmented manually. We modeled infarct volume as a continuous variable using restricted cubic splines and tested for an interaction with treatment allocation in mixed effects logistic regression.

**Results:**

We included 3572 participants (mean age = 78 ± 10 years, 45% female), 98.6% of the main trial population. The effect of early versus delayed anticoagulation did not vary with infarct volume (p_interaction_ = 0.18). Rates of the primary outcome were 17/568 (3.0%) and 12/599 (2.0%) for early versus delayed initiation with infarcts of 0–5 mL; 6/220 (2.7%) and 11/229 (4.8%) with infarcts of 5–10 mL; 13/258 (4.6%) and 10/283 (3.5%) with infarcts of 10–25 mL; 6/145 (4.1%) and 8/145 (5.5%) with infarcts of 25–50 mL; 1/93 (1.1%) and 7/94 (7.4%) with infarcts of >50 mL; and 14/481 (2.9%) and 10/430 (2.2%) in participants with no infarct visible on clinically acquired brain imaging. Corresponding odds ratios and 95% confidence intervals were 1.52 (0.71–3.20), 0.55 (0.20–1.51), 1.29 (0.55–3.00), 0.74 (0.25–2.21), 0.13 (0.02–1.11), and 1.25 (0.55–2.86), respectively. There were no increased rates of symptomatic ICH with respect to anticoagulation timing for those with large infarcts (>25 mL); there were 3/238 (1.3%) events in the early group and 5/239 (2.1%) in the delayed group.

**Conclusion:**

The treatment effect of early anticoagulation with a DOAC in acute ischemic stroke associated with atrial fibrillation was not modified by infarct volume. Adverse outcomes were not increased with early anticoagulation in people with larger infarcts. Our results provide no evidence that anticoagulation initiation should be delayed beyond 4 days on the basis of infarct size.

## Graphical abstract

**Graphical abstract:**
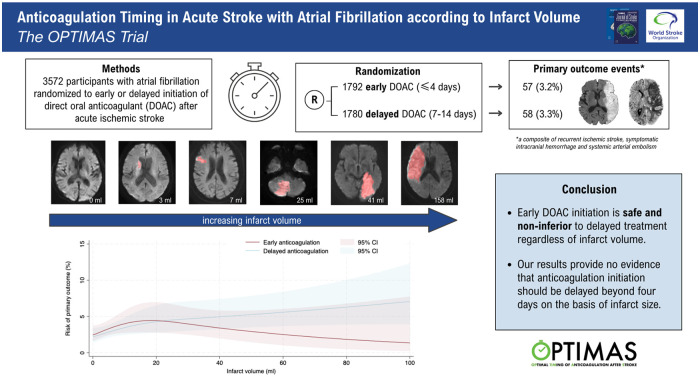

## Introduction

When to start anticoagulation after an acute ischemic stroke in patients with atrial fibrillation (AF) is a frequent dilemma in stroke medicine. Historically, anticoagulation has been delayed by up to 2 weeks due to concern about the risk of hemorrhagic transformation of the infarct; however, several large randomized controlled trials—TIMING, ELAN, OPTIMAS and START—recently found similar rates of ischemic and hemorrhagic events in those initiating anticoagulation with a direct oral anticoagulant (DOAC) soon after stroke to those initiating anticoagulation later.^1–4^ Recently, the CATALYST collaboration, an individual patient data meta-analysis including participants from these trials, demonstrated superiority of early DOAC initiation in preventing recurrent stroke within 30 days.^5^

There nevertheless remains concern that DOAC initiation should be delayed in patients with larger infarcts, who are at greater risk of hemorrhagic transformation,^6^ leading to recommendations to delay anticoagulation in patients with clinically severe stroke.^7^ Although anticoagulation timing trials to date have not shown treatment effect modification by clinical stroke severity, this is a potentially inaccurate proxy for infarct volume. Randomized data on whether infarct size itself modifies the effect of early anticoagulation are limited to a secondary analysis of ELAN, in which rates of the primary outcome of all-cause stroke, systemic embolism, major extracranial bleeding, and vascular death within 30 days did not differ between early and late treatment groups, whether infarct size was classified as minor, moderate, or major, using a semiquantitative visual rating method.^8^ However, infarct volume was not directly measured, classification was influenced by infarct arterial territory and number, and the study protocol varied anticoagulation timing systematically according to infarct classification.

In this study, we did a prespecified neuroimaging-based secondary analysis of the OPTIMAS clinical trial—the largest trial of the timing of anticoagulation initiation after AF-associated ischemic stroke to date—investigating whether infarct volume, measured precisely using segmentation, modifies the treatment effect of early anticoagulation, with the potential for harm in patients with larger infarcts. We also investigated whether treatment effect varied according to infarct location.

## Methods

### Study design and participants

We did a prespecified secondary analysis of OPTIMAS, a randomized, controlled, parallel-group, open label with blinded-endpoint adjudication (PROBE) trial investigating the effects of early versus delayed anticoagulation with a DOAC in participants with acute ischemic stroke and AF. The study protocol,^9^ statistical analysis plan,^10^ protocol for this prespecified analysis,^10^ and main trial results have already been published.^3^ Participants were recruited at 100 hospitals in the United Kingdom (Supplemental Table S1). Key inclusion criteria were a clinical diagnosis of acute ischemic stroke, a known history of AF or new diagnosis of AF confirmed by electrocardiogram (ECG), and eligibility for anticoagulation with a DOAC. All participants underwent neuroimaging to exclude intracerebral hemorrhage as the cause of stroke and to assess for hemorrhagic transformation according to Heidelberg criteria.^11^ Participants with hemorrhagic transformation remained eligible unless graded locally as parenchymal hematoma 2 (PH2) or remote from the qualifying infarct. Full eligibility criteria have been published elsewhere.^3,9^ Results are reported according to CONSORT guidelines.

### Randomization and masking

Participants were randomized 1:1 to early DOAC initiation (within 4 days of stroke onset) or delayed DOAC initiation (at 7–14 days). The exact time of initiation within the allocated window was chosen by the treating clinician. Randomization was stratified by stroke severity as measured by NIHSS. Participants and treating teams were not blinded to allocation, but outcome events were adjudicated by an independent panel of expert stroke clinicians, blinded to allocation.

### Outcomes

The primary outcome was a composite of recurrent ischemic stroke, symptomatic intracranial hemorrhage (ICH), unclassified stroke, or systemic arterial embolism within 90 days of randomization. Prespecified secondary outcomes for this analysis were recurrent ischemic stroke and symptomatic ICH, including extradural, subdural, subarachnoid, and intracerebral hemorrhage, and hemorrhagic transformation of the qualifying infarct. Our main exposure of interest for this secondary analysis was the volume of the acute infarct; we also investigated outcomes according to the ELAN stroke size classification and according to infarct location.^8^

### Study procedures

The central study team, blinded to treatment allocation, collected pseudonymized brain imaging studies related to the qualifying acute stroke or performed during follow-up. The study protocol did not specify imaging to be done at any specific timepoints, and acquired studies were done for clinical reasons, including outcome events. Imaging was checked for completeness and quality by trained researchers (P.S.N. and J.G.B.), and scans of non-diagnostic quality excluded. When both diagnostic-quality magnetic resonance imaging (MRI) and computed tomography (CT) studies were available, we used MRI owing to its greater sensitivity and accuracy. When multiple eligible scans of the same modality and adequate technical quality were available, the last scan before DOAC administration was used for analysis.

For the quantitative assessment of infarcts on MRI, we used a deep learning lesion segmentation model for diffusion-weighted imaging (DWI) developed, validated out of sample, and described in detail elsewhere.^12^ All model-derived lesion masks were reviewed and—if required—refined by a trained reader under the supervision of an experienced neuroradiologist specializing in stroke imaging with more than 15 years of experience (DD). Just 4.7% of lesion masks needed refinement.

For participants without available MRI, infarcts were segmented on CT by trained researchers (P.S.N., J.L., T.A., and R.M.), under the supervision of DD, using semi-automated techniques in ITKSnap version 3.6.0. To minimize inter-rater variability, all raters first rated a training set of 30 images labeled by D.D. Intra-class correlation coefficients of 0.88 to 0.97 were achieved. Uncertain cases were reviewed by D.D. Participants with no visible brain infarct were treated as having an infarct volume of 0 mL.

The recently developed ELAN stroke severity scale was used to assess infarct volume qualitatively. Inter-rater reliability was assessed using Krippendorff’s alpha with all raters scoring 1.00 for ELAN classification.

*Supplemental Methods* give additional information on imaging analysis procedures. Supplemental Figure S1 shows example segmentations.

### Statistical analysis

The target sample size for the main OPTIMAS trial was 3478 participants, intended to provide 80% power to show non-inferiority of early anticoagulation based on a non-inferiority margin of 2%, a primary outcome event rate of 4·3%, and a two-sided significance level of 5%. At the recommendation of trial steering and independent data monitoring committees, recruitment continued for as long as study funding allowed, leading to a larger sample size. In this secondary analysis, we included all participants with a diagnostic-quality CT or MRI and follow-up data for the primary study outcome. Analyses followed the modified intention-to-treat principle, whereby participants inadvertently enrolled without confirmed AF and ischemic stroke were excluded.

To assess whether each neuroimaging variable modified the treatment effect of the OPTIMAS trial intervention, we fitted binary mixed effects logistic regression models with the variable of interest and allocated treatment as interaction variables. We treated infarct volume as a continuous variable. To allow for non-linear effects, we investigated modeling infarct volume using a restricted cubic spline, with knot number selected to minimize the Akaike information criterion (AIC) and knots positioned according to expert recommendations.^13^ The optimal model, a restricted cubic spline with three knots set at the 10th, 50th, and 90th centiles, was used in subsequent analysis (ΔAIC = −2.04 compared with the untransformed model with respect to the primary outcome). We treated the ELAN classification and infarct location as categorical variables. Study sites were included as random intercept terms.

Statistical analyses were performed by P.S.N., J.L., and J.G.B. using Stata (version 18; StataCorp LLC, College Station, TX).

## Results

**Figure 1.**
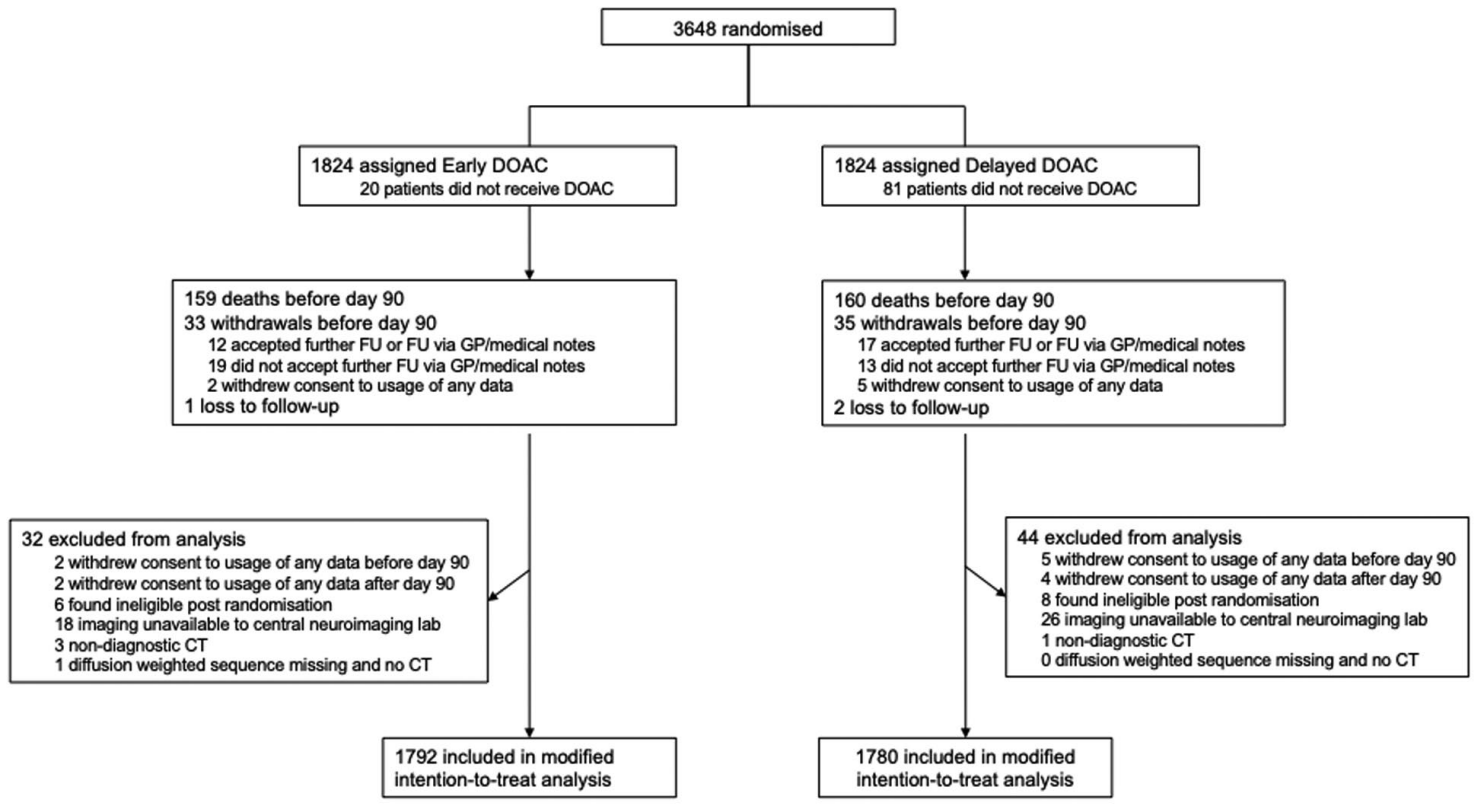
CONSORT diagram showing patient flow through the trial for this OPTIMAS sub-analysis. *DOAC* direct oral anticoagulant.

OPTIMAS recruited 3648 participants between 4 July 2019 and 31 January 2024, with last follow-up on 10 July 2024. A total of 3621 participants were included in the modified intention-to-treat population. For this analysis, a further 49 participants were excluded because of missing or non-diagnostic imaging data, leaving 3572 participants. Figure 1 shows participant flow through the trial, and Supplemental Table S2 shows that there were no substantial differences in the characteristics of included and excluded participants. Participant characteristics were well-balanced between treatment groups, including demographics, co-morbidities, infarct volume and distribution (Table 1). The mean (SD) age in the early DOAC group was 77.9 (9.9) years and 78.0 (9.9) years in the delayed group. During the 90-day follow-up period, 115 participants experienced a primary outcome event, including 83 recurrent ischemic strokes and 23 symptomatic ICH.

**Table 1.** Baseline characteristics according to allocated treatment group.

	Early DOAC (n = 1792)	Delayed DOAC (n = 1780)	All participants (n = 3572)
Age: years, mean (SD)	77.9 (9.9)	78.0 (9.9)	78.0 (9.9)
Sex: female	799 (44.6%)	821 (46.1%)	1620 (45.4%)
Ethnicity			
White	1670 (93.2%)	1677 (94.2%)	3347 (93.7%)
Black British, African, or Caribbean	31 (1.7%)	27 (1.5%)	58 (1.6%)
South Asian	30 (1.7%)	30 (1.7%)	60 (1.7%)
East Asian or Southeast Asian	23 (1.3%)	16 (0.9%)	39 (1.1%)
Mixed ethnicity; other; not disclosed or missing	38 (2.1%)	30 (1.7%)	68 (1.9%)
Hypertension	1187 (66.2%)	1215 (68.3%)	2402 (67.2%)
Diabetes	384 (21.4%)	367 (20.6%)	751 (21.0%)
Hypercholesterolemia	612 (34.2%)	559 (31.4%)	1171 (32.8%)
Chronic kidney disease	266 (14.8%)	268 (15.1%)	534 (14.9%)
Myocardial infarction	161 (9.0%)	168 (9.4%)	329 (9.2%)
Previous ischemic stroke	291 (16.2%)	241 (13.5%)	532 (14.9%)
Previous intracranial hemorrhage	35 (2.0%)	28 (1.6%)	63 (1.8%)
Known cognitive impairment	117 (6.5%)	123 (6.9%)	240 (6.7%)
Current or former smoker	640 (37.3%)	639 (37.3%)	1279 (37.3%)
Alcohol intake > 14 units/week	210 (11.7%)	188 (10.6%)	398 (11.1%)
Previous anticoagulation	632 (35.3%)	631 (35.4%)	1263 (35.4%)
Vitamin K antagonist	59 (9.3%)	51 (8.1%)	110 (8.7%)
DOAC	573 (90.7%)	580 (91.9%)	1153 (91.3%)
Previous antiplatelet use	211 (11.8%)	189 (10.6%)	400 (11.2%)
IV thrombolysis	416 (23.2%)	370 (20.8%)	786 (22.0%)
Endovascular treatment	131 (7.3%)	132 (7.4%)	263 (7.4%)
NIHSS on admission, median (IQR)	6 (3–11)	5 (3–10)	5 (3–10)
NIHSS at randomization			
0–4	1032 (57.6%)	1036 (58.2%)	2068 (57.9%)
5–10	499 (27.8%)	494 (27.8%)	993 (27.8%)
11–15	145 (8.1%)	130 (7.3%)	275 (7.7%)
16–21	86 (4.8%)	87 (4.9%)	173 (4.8%)
>21	30 (1.7%)	33 (1.9%)	63 (1.8%)
NIHSS at randomization, median (IQR)	4 (2–7)	4 (2–7)	4 (2–7)
Pre-stroke mRS, median (IQR)	0 (0–2)	0 (0–2)	0 (0–2)
Infarct volume (mL)			
Median (IQR)	2.7 (0.0–12.7)	3.0 (0.1–13.1)	2.8 (0.0–12.9)
Mean (SD)	11.7 (24.2)	11.6 (23.6)	11.7 (23.8)
Infarct volume determined using MR	525 (29.3%)	569 (32.0%)	1094 (30.6%)
Infarct volume determined using CT	1267 (70.7%)	1211 (68.0%)	2478 (69.4%)
Hours between onset and imaging (median, IQR)	14.0 (4.0–27.0)	13.0 (5.0–27.0)	13.0 (5.0–27.0)
ELAN classification			
Minor	732 (40.8%)	709 (39.8%)	1441 (40.3%)
Moderate	814 (45.4%)	823 (46.2%)	1637 (45.8%)
Major	246 (13.7%)	248 (13.9%)	494 (13.8%)
Infarct arterial territory			
Anterior circulation	997 (55.6%)	1033 (58.0%)	2030 (56.8%)
Posterior circulation	218 (12.2%)	221 (12.4%)	439 (12.3%)
Multiterritory	96 (5.4%)	96 (5.4%)	192 (5.4%)
No visible infarct	481 (26.8%)	430 (24.2%)	911 (25.5%)

*DOAC* direct oral anticoagulant; *NIHSS* National Institute for Health Stroke Scale; *BP* blood pressure; *mRS* modified Rankin Score.

The median (IQR) interval between stroke onset and diagnostic imaging was 13 (5–27) h, and this was well-balanced between groups (Table 1). An infarct was visible on the clinically acquired brain imaging collected before randomization in 1063/1094 (97.2%) participants assessed using MRI, and 1596/2748 (64.5%) participants assessed using CT only. The characteristics of participants whose infarct volumes were determined using CT and MRI are compared in Supplemental Table S3. Overall, 2661/3572 (74.5%) participants had a visible infarct. In these participants, the median infarct volume was 6.5 mL (IQR = 1.8–18.3). Including participants without visible infarcts, the median volume was 2.8 mL (IQR = 0.0–12.9). In the 1063 participants with an infarct segmented on MRI, the median infarct volume was 3.3 mL (IQR = 1.0–9.9); for the 1598 participants with an infarct segmented on CT, it was 9.6 mL (IQR = 3.2–24.3). Table 2 shows the distribution of participants into groups defined by infarct volume and outcome event rates according to treatment allocation in each group. Supplemental Table S4 shows baseline characteristics according to infarct volume.

**Table 2.** Participants and 90-day event rates according to infarct volume.

Infarct volume	Participants, N (%)	Primary outcome events		Ischemic stroke events		Symptomatic ICH events	
		N	% (95% CI)	N	% (95% CI)	N	% (95% CI)
No visible infarct							
All participants	911 (25.5)	24	2.6 (1.7–3.9)	20	2.2 (1.3–3.4)	1	0.1 (0.0–0.6)
Early DOAC	481 (13.5)	14	2.9 (1.6–4.8)	11	2.3 (1.1–4.1)	1	0.2 (0.0–1.2)
Delayed DOAC	430 (12.0)	10	2.3 (1.1–4.2)	9	2.1 (1.0–4.0)	0	0.0 (0.0–0.9)
Infarct < 5 mL							
All participants	1167 (32.7)	29	2.5 (1.7–3.6)	22	1.9 (1.2–2.9)	4	0.3 (0.1–0.9)
Early DOAC	568 (15.9)	17	3.0 (1.7–4.8)	14	2.5 (1.3–4.1)	2	0.4 (0.0–1.3)
Delayed DOAC	599 (16.8)	12	2.0 (1.0–3.5)	8	1.3 (0.6–2.6)	2	0.3 (0.0–1.2)
5–10 mL							
All participants	449 (12.6)	17	3.8 (2.2–6.1)	12	2.7 (1.4–4.7)	3	0.7 (0.1–2.0)
Early DOAC	220 (6.2)	6	2.7 (1.0–5.9)	3	1.4 (0.3–4.0)	2	0.9 (0.1–3.3)
Delayed DOAC	229 (6.4)	11	4.8 (2.4–8.6)	9	3.9 (1.8–7.5)	1	0.4 (0.0–2.4)
10–25 mL							
All participants	568 (15.9)	23	4.0 (2.6–6.1)	15	2.6 (1.5–4.4)	7	1.2 (0.5–2.5)
Early DOAC	285 (8.0)	13	4.6 (2.4–7.8)	10	3.5 (1.7–6.5)	3	1.1 (0.2–3.1)
Delayed DOAC	283 (7.9)	10	3.5 (1.7–6.5)	5	1.8 (0.6–4.1)	4	1.4 (0.4–3.6)
25–50 mL							
All participants	290 (8.1)	14	4.8 (2.6–8.1)	9	3.1 (1.4–5.9)	5	1.7 (0.6–4.0)
Early DOAC	145 (4.0)	6	4.1 (1.5–9.0)	4	2.8 (0.8–7.1)	2	1.4 (0.2–5.0)
Delayed DOAC	145 (4.0)	8	5.5 (2.3–10.9)	5	3.4 (1.1–8.0)	3	2.1 (0.4–6.0)
>50 mL							
All participants	187 (5.2)	8	4.3 (1.8–8.4)	5	2.7 (0.9–6.2)	3	1.6 (0.3–4.7)
Early DOAC	93 (2.6)	1	1.1 (0.0–6.0)	0	0.0 (0.0–4.0)	1	1.1 (0.0–6.0)
Delayed DOAC	94 (2.6)	7	7.4 (3.0–15.3)	5	5.3 (1.7–12.4)	2	2.1 (0.3–7.7)

*DOAC* direct oral anticoagulant, *ICH* intracranial hemorrhage.

### Primary outcome according to infarct volume

**Figure 2.**
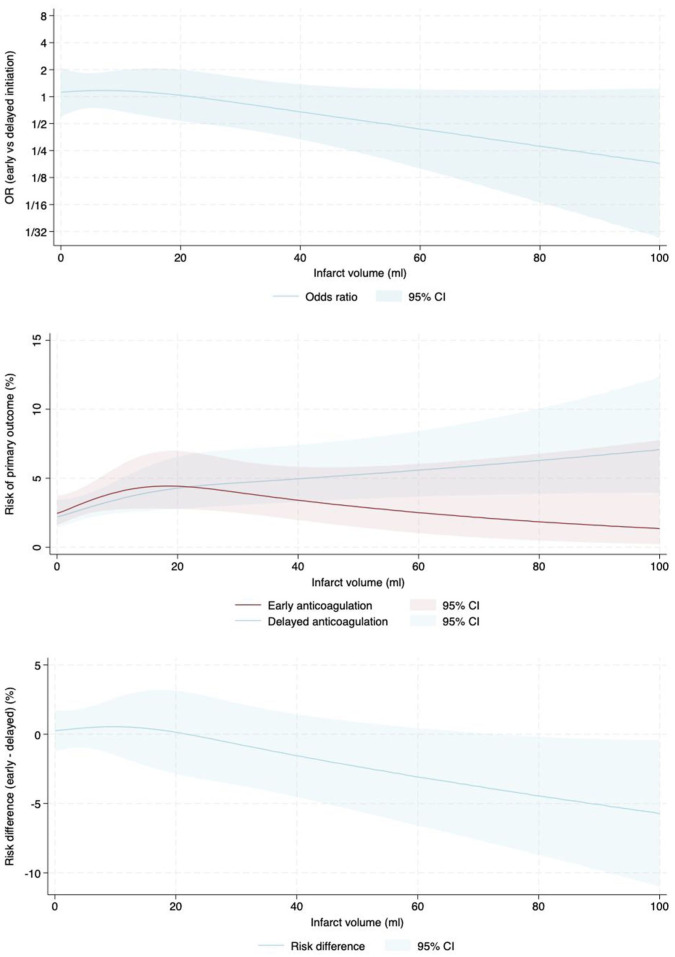
Treatment effect of early versus delayed anticoagulation with respect to the composite primary outcome according to infarct volume. *DOAC* direct oral anticoagulant. For display purposes, the *x*-axis is truncated at 100 mL. Only 46 participants had an infarct volume >100 mL, and only one primary outcome event occurred in those participants.

**Figure 3.**
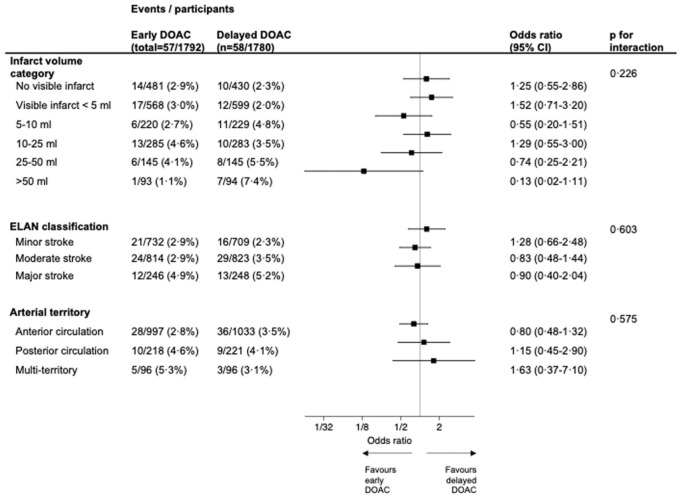
Subgroup analysis for risk of the primary composite outcome according to treatment allocation, infarct volume categories, ELAN classification and arterial territory. *DOAC* direct oral anticoagulant.

We found no evidence that the effect of early versus delayed anticoagulation varied significantly across the range of observed infarct volumes with respect to the primary outcome (p_interaction_ = 0.18). Figure 2 shows the estimated odds ratios and risk difference between treatment groups according to infarct volume for the primary outcome. Figure 3 shows illustrative odds ratios according to infarct volume categories. Numerically, the risk of the primary outcome in the early group was very similar to or lower than that in the delayed group across the range of infarct volumes studied. The greatest numerical difference between treatment groups was observed in participants with large infarcts (>50 mL), in whom the primary outcome occurred in 1/93 participants in the early group (1.1%, 95% CI = 0.0–5.9) and 7/94 participants in the delayed group (7.4%, 95% CI = 3.1–14.7), although these estimates were imprecise and based on a small number of participants and outcome events.

### Secondary outcomes according to infarct volume

We found no evidence of heterogeneity in treatment effect according to infarct volume for recurrent ischemic stroke (p_interaction_ = 0.34) or symptomatic ICH (p_interaction_ = 0.50). Supplemental Figures S2 and S3 show the estimated odds ratios for early versus delayed anticoagulation according to infarct volume for each outcome, with associated risks in each treatment group and the difference in risk between groups. As infarct volume increased, there were more symptomatic ICH events overall, but these were balanced between treatment groups, with no excess in those allocated to early anticoagulation (Table 2).

### ELAN classification

By ELAN classification, 1441/3572 (40.3%) participants had a minor infarct, 1637/3572 (45.8%) a moderate infarct, and 494/3572 (13.8%) a major infarct. The overall rate of the primary outcome increased with infarct size: 2.6% with a minor infarct, 3.2% with a moderate infarct, and 5.1% with a major infarct. However, we found no evidence that the treatment effect of early anticoagulation was modified by ELAN classification for the primary outcome (p = 0.603, Figure 3) or secondary outcomes (Supplemental Table S4).

### Infarct location

Of 2661 participants with a visible infarct, 2030 (77.7%) affected the anterior circulation only, 439 (16.5%) the posterior circulation only, and 192 (7.2%) involved both simultaneously. Supplemental Figure S4 shows their detailed anatomical distribution. We found no evidence of a difference in treatment effect according to infarct location for the primary outcome (p_interaction_ = 0.575) (Figure 3) or secondary outcomes (Supplemental Table S4).

### Timing of DOAC initiation according to infarct volume

The median interval (IQR) between onset and DOAC initiation was 3 days (3–4) in the early group and 8 days (7–9) in the delayed group. In the delayed group, the interval was slightly longer for the largest infarct volume category, at 9 days (8–12); otherwise, there was little variation in actual DOAC initiation time according to infarct volume (Supplemental Table S5).

### Sensitivity analysis

We did two prespecified sensitivity analyses with respect to the primary study outcome. First, to address possible error introduced by the underestimation of infarct volume in participants for whom only a very early CT scan was available, we repeated our analysis omitting the 662 participants in whom infarct volume was measured using a CT scan within 6 h of onset. Second, we repeated our analysis, including only participants in whom infarct volume was measured using MRI, giving high sensitivity for small and very early infarcts. We did a third post hoc sensitivity analysis, omitting participants in whom no infarct was visible on the clinical brain imaging undertaken. As in our main analysis, we found no evidence that the treatment effect varied according to infarct size when omitting participants with early CT (p_interaction_ = 0.20, Supplemental Figure S5) or no visible infarct (p_interaction_ = 0.20), or including only participants with MRI (p_interaction_ = 0.67).

## Discussion

In this prespecified secondary analysis of the OPTIMAS trial, we did not find evidence that the impact of early DOAC initiation varies by acute infarct volume, visual size classification, or location. We did not find excess symptomatic ICH events in participants with larger infarcts allocated to early anticoagulation, providing reassurance that early anticoagulation with a DOAC might reasonably be considered in most patients within 4 days of the onset of AF-associated ischemic stroke, including those with larger infarcts and infarcts in the posterior circulation. Although, in line with the overall trial results, we did not find evidence that early DOAC administration significantly reduced the rate of the primary outcome in the subgroups tested, early initiation, irrespective of imaging features, may have other practical benefits, including reduced use of interval neuroimaging and greater adherence to DOAC treatment following discharge.^14^ The results of this study should be considered in the context of the CATALYST individual patient data meta-analysis, which showed a small but statistically significant benefit of early anticoagulation within 4 days of onset with respect to recurrent stroke of any cause within 30 days.^5^ This suggests that early anticoagulation should, in general, be preferred to delayed anticoagulation, and our results provide no evidence that it is necessary to deviate from this policy on the basis of infarct size.

An important strength of our study is that we measured infarct volume directly. We chose this approach because we considered the volume of infarcted brain tissue to be the most biologically plausible predictor of hemorrhagic transformation risk, and measuring infarct size as a continuous variable would give the greatest statistical power to detect an interaction if present. Our analysis also allowed for a non-linear interaction between infarct volume and treatment allocation. Although manually segmenting infarcts is unlikely to be feasible in current clinical practice, advances in automated segmentation may soon make this possible. However, our results suggest that measuring infarct volume, whether precisely or through a clinically applicable visual approximation as in ELAN, might be unnecessary when deciding upon anticoagulation timing in most cases.

Additional methodological strengths of our study include the recruitment of a multicenter study sample from 100 centers, with baseline characteristics similar to unselected stroke patients in the United Kingdom, including a median NIHSS at presentation of 6 (IQR = 3–11), greater than that observed in the national UK SSNAP audit (4, IQR = 2–9) and only slightly lower than that of patients with AF in the Swiss Stroke Registry (7, IQR = 3–15).^15,16^ We obtained high rates of neuroimaging data availability and follow-up, including 98.6% of trial participants in this secondary analysis. Neuroimaging measures were rated centrally by trained raters, who obtained excellent inter-rater reliability in training data sets. All outcome events were adjudicated centrally, blinded to treatment allocation and before rating of baseline neuroimaging data.

We acknowledge some limitations. This was a secondary analysis of a trial that was powered only for its prespecified primary analysis, not to detect interactions with subgroups. Outcome event rates were lower than anticipated; symptomatic ICH in particular was infrequent. Although the clinical characteristics of participants, including stroke severity, were similar to those of unselected stroke patients, we included relatively few participants with very large infarcts—187/3572 (5.2%) participants had an infarct larger than 50 mL and 46/3572 (1.3%) larger than 100 mL—possibly indicating reluctance by clinicians to enroll patients they considered “high risk” on the basis of imaging findings. Consequently, our estimates were imprecise for larger infarcts and may not generalize to patients with very large infarcts (e.g. >100 mL) who were not well represented in this study.

We used brain imaging performed in routine clinical practice, so imaging modality and timing were not standardized. This may have introduced measurement error. Notably, very early CT (less than 6 h from stroke onset) may underestimate final infarct volume or fail to detect small infarcts. To mitigate this, while pragmatically reflecting clinical practice, we used the last available scan before DOAC initiation and preferred diffusion-weighted MRI if available, on which abnormal signal can be observed minutes from stroke onset. Despite this, around one-quarter of participants had no detectable infarct on available imaging. However, we found no evidence of treatment effect modification by infarct size in sensitivity analyses excluding very early CT, restricted to patients with diffusion-weighted MRI, or omitting participants with no visible infarct.

Other limitations of our study include the enrolment of relatively few participants of Black, Asian, and other non-White ethnicities, at least in part reflecting the epidemiology of an older population in the United Kingdom defined by the presence of AF.^17^ This is relevant given the recognized ethnic differences between Asians and Europeans for ischemic stroke and ICH.^18^ Finally, the exact timing of anticoagulation initiation within the allocated treatment window was determined by the treating physician and may have been influenced by neuroimaging findings including infarct size, although we found limited variation in actual anticoagulation timing across infarct volume categories.

In summary, we did not find evidence that brain imaging findings on infarct size or location should influence the timing of anticoagulation in most patients with AF and recent stroke. This finding is consistent with the results of the ELAN neuroimaging substudy.^8^ Larger studies are needed to more precisely estimate the effect of early anticoagulation in patients with very large infarcts. A dedicated randomized controlled trial in such patients would require a large sample size and face considerable practical challenges, but a pooled analysis of individual patient imaging data with START and ELAN is planned within the CATALYST collaboration as a next step. Clinicians should nevertheless be reassured that the available evidence from two large randomized trials does not support delaying anticoagulation on the basis of infarct size.

## Supplemental Material

sj-pdf-1-wso-10.1177_17474930261441297 – Supplemental material for Early versus delayed anticoagulation in acute ischemic stroke with atrial fibrillation according to infarct volume and location: A prespecified subgroup analysis of the OPTIMAS randomized controlled trial Supplemental material, sj-pdf-1-wso-10.1177_17474930261441297 for Early versus delayed anticoagulation in acute ischemic stroke with atrial fibrillation according to infarct volume and location: A prespecified subgroup analysis of the OPTIMAS randomized controlled trial by Philip S Nash, Jonathan G Best, James Lyon, James K Ruffle, Thanyalak Amornpojnimman, Rom Mendel, Chris Foulon, Hakim-Moulay Dehbi, Norin Ahmed, Liz Arram, Maryam Balogun, Kate Bennett, Ekaterina Bordea, Emilia Caverly, Marisa Chau, Hannah Cohen, Mairead Cullen, Caroline J Doré, Stefan T Engelter, Robert Fenner, Gary A Ford, Aneet Gill, Rachael Hunter, Martin James, Archana Jayanthi, Gregory YH Lip, Sue Massingham, Macey L Murray, Iwona Mazurczak, Amalia Ndoutoumou, Bo Norrving, Jenny Philip, Hannah Sims, Nikola Sprigg, Tishok Vanniyasingam, Parashkev Nachev, Nick Freemantle, David Doig and David J Werring in International Journal of Stroke

sj-png-2-wso-10.1177_17474930261441297 – Supplemental material for Early versus delayed anticoagulation in acute ischemic stroke with atrial fibrillation according to infarct volume and location: A prespecified subgroup analysis of the OPTIMAS randomized controlled trial Supplemental material, sj-png-2-wso-10.1177_17474930261441297 for Early versus delayed anticoagulation in acute ischemic stroke with atrial fibrillation according to infarct volume and location: A prespecified subgroup analysis of the OPTIMAS randomized controlled trial by Philip S Nash, Jonathan G Best, James Lyon, James K Ruffle, Thanyalak Amornpojnimman, Rom Mendel, Chris Foulon, Hakim-Moulay Dehbi, Norin Ahmed, Liz Arram, Maryam Balogun, Kate Bennett, Ekaterina Bordea, Emilia Caverly, Marisa Chau, Hannah Cohen, Mairead Cullen, Caroline J Doré, Stefan T Engelter, Robert Fenner, Gary A Ford, Aneet Gill, Rachael Hunter, Martin James, Archana Jayanthi, Gregory YH Lip, Sue Massingham, Macey L Murray, Iwona Mazurczak, Amalia Ndoutoumou, Bo Norrving, Jenny Philip, Hannah Sims, Nikola Sprigg, Tishok Vanniyasingam, Parashkev Nachev, Nick Freemantle, David Doig and David J Werring in International Journal of Stroke
