# Supplementary material for: Early versus delayed anticoagulation in acute ischemic stroke with atrial fibrillation according to infarct volume and location: A prespecified subgroup analysis of the OPTIMAS randomized controlled trial
Source: Int J Stroke. 2026 Mar 30;21(7):956–67. doi: 10.1177/17474930261441297 (PMC13392158; doi:10.1177/17474930261441297)
Supplement: sj-pdf-1-wso-10.1177_17474930261441297 – Supplemental material for Early versus delayed anticoagulation in acute ischemic stroke with atrial fibrillation according to infarct volume and location: A prespecified subgroup analysis of the OPTIMAS randomized controlled trial [file sj-pdf-1-wso-10.1177_17474930261441297.pdf]

Supplement

Table of contents

Page 2	Supplementary Methods
Page 3	Table S1: OPTIMAS trial sites and investigators
Page 7	Table S2: Characteristics of included and excluded participants
Page 8	Table S3: Baseline characteristics according to infarct volume categories
Page 10	Table S4: Secondary outcomes according to infarct characteristics
Page 11	Table S5: DOAC initiation timing according to treatment allocation and subgroups
Page 12	Figure S1: Example segmentations
Page 13	Figure S2: Treatment effect of early anticoagulation with respect to recurrent ischaemic stroke by infarct volume
Page 14	Figure S3: Treatment effect of early anticoagulation with respect to symptomatic intracranial haemorrhage by infarct volume
Page 15	Figure S4: Heatmaps showing voxel-based density of infarcted tissue according to treatment allocation
Page 16	Figure S5: Sensitivity analysis omitting participants with infarct volume measured on CT within six hours of onset
Page 17	Supplementary References

Supplementary Methods

Preprocessing
Clinically-acquired brain imaging was supplied by sites in DICOM format, and was converted to NIfTI format prior to analysis using the dicom2nifti and dcm2niix open-source software packages.

MRI segmentation model
In brief, the imaging segmentation model uses the Swin UNet TRansformer architecture with a custom loss function that optimises quantification of the high-resolution anatomy and presence or absence of infarcts. This provides fidelity in delineating both single and multifocal infarcts. Lesion segmentation was performed in Montreal Neurological Institute (MNI) stereotactic space, at 2x2x2mm resolution, following non-linear registration with SPM12 with parameters derived from the b0 image and applied to the b1000 image (1,2). Subsequent analysis was performed on the resultant binary lesion mask transformed into native imaging space with the inverse deformation field obtained from the non-linear registration step. The model was trained on neuroimaging data from 10,463 patients, which included 3563 infarct-positive diffusion-weighted imaging (B1000) cases acquired as part of routine clinical care at a tertiary neuroscience centre, and 6900 diffusion-negative controls. The model was specifically evaluated for clinical relevance on a large clinical dataset, with equitable performance across patient demographics and all vascular territories.

Manual segmentation procedures
When manually segmenting CT infarcts, raters used standard “stroke windows” (level 40, width 40) to identify the lesion, and if needed these were adjusted manually to optimally delineate margins. If available, we segmented the infarct on thick (usually 5mm) axial slices, but thin slices (0·5 – 1mm) were used if necessary for difficult cases.

Table S1: Complete list of OPTIMAS trial sites and investigators, listed in descending order of the number of participants recruited, with Principal Investigators denoted by PI

Site	Participants	Investigators
University Hospital of Wales, Cardiff	175	Dr B Jelley PI, Dr T Hughes, M Evans, D G Esteban, L Knibbs, L Broad, R Price, L H Griebel, S Hewson
Royal Bournemouth Hospital, Bournemouth	166	Dr K Thavanesan PI, L Mallon, A Smith, M White
St Georges Hospital, London	158	Dr L Zhang PI, Dr B Clarke, Dr Y Abousleiman, L Binnie, C H Sim, M Castanheira
University College London Hospitals NHS Foundation Trust, London	147	Dr F Humphries PI, S Obarey, S Feerick, Y C Lee, A Lewis, R Muhammad, N Francia, N Atang, A Banaras, M Marinescu
Royal Stoke University Hospital, Stoke	131	Dr P Ferdinand PI, R Varquez, I Ponce, S Saxena
Addenbrooke's Hospital, Cambridge	111	Dr E O'Brien PI, Dr J D Reyes, J Mitchell-Douglas, J Francis
Charing Cross Hospital, London	93	Dr S Banerjee PI, V Dave, S Mashate, T Patel
Luton & Dunstable University Hospital, Luton	88	Dr L Sekaran PI, Dr W Murad, Dr A Asaipillai, Dr S Sakthivel, T L Margaret, J Angus, L Reid, C Fornolles, S Sundayi, L Poolon, F Justin, S Hunte
Watford General Hospital, Watford	84	Dr M Bhandari PI, S Sundayi, J Kho
Victoria Hospital, Fife	79	Dr V Cvoro PI, Dr R Parakramawansha, M Couser, H Hughes
Royal Hallamshire Hospital, Sheffield	75	Dr A Naqvi PI, Dr K Harkness, E Richards, J Howe, C Kamara, J Gardner
John Radcliffe Hospital, Oxford	75	Dr H Bains PI, R Teal, J Joseph, J Benjamin
James Cook University Hospital, Middlesborough	73	Dr S Al-Hussayni PI, Dr G Thomas, F Robinson, L Dixon
Morriston Hospital, Swansea	71	Dr M Krishnan PI, Dr P Slade, Dr T Anjum, S Storton
Royal Cornwall Hospital, Truro	68	Dr K Adie PI, K Northcott, K Morgan, E Williams
Leighton Hospital, Crewe	67	Dr H Chandrashekar PI, H Maguire, C Gabriel, D Maren, H David, S Clarke
Royal Berkshire, Reading	66	Dr K Nagaratnam PI, Dr V Nelatur, N Mannava, L Blasco
Northwick Park Hospital, Harrow	62	Dr J Devine PI, Dr R Bathula, P Gopi, N Mehta S Sreedevi Raj
King's College Hospital NHS Foundation Trust, London	60	Dr J Teo PI, Dr L Sztriha, Dr Y Mah, Dr S Ankolekar, B Sari, M Tibajai, A Morgan, M Recaman, S Bayhonan, C Belo
Royal Devon & Exeter Hospital, Exeter	60	Prof M James PI, S Finch, S Keenan, A Bowring
Nottingham University Hospitals NHS Trust, Nottingham	57	Dr A Shetty PI, Dr S Chan, L Gray
Royal London Hospital, London	53	Dr T Harrison PI, Dr O Spooner, E Kinsella-Perks, E Erumere, B Sanders
Queen Elizabeth University Hospital, Birmingham	48	Dr D Sims PI, Dr M Willmot, Dr E Littleton, E Spruce, L Moody, C Sheriden, S Luxmore-Brown, A Neal, S Beddows
Wycombe Hospital, High Wycombe	45	Dr M A Tuna PI, Dr A Misra, R Penn, S Mariampillai
North Tees Hospital, Stockton-on-Tees	45	Dr I Anwar PI, Dr A Annamalai, Dr S Whitehouse, L Shepherd, E Siddle
Countess of Chester Hospital, Chester	44	Dr K Chatterjee PI, S Leason, A Davies
Southampton General Hospital, Southampton	42	Dr R Marigold PI, S Frank, A Baird, T Hannam-Penfold, L Inacio, S Smith

Leicester Royal Infirmary, Leicester	41	Dr D Eveson PI, Dr K Musarrat, S Khan, T Harris
Ipswich Hospital, Ipswich	41	Dr M R Chowdhury PI, Dr S Alam, Dr E Jamieson, Dr E Anyankpele, Dr F Al Shalchi, V Rivers, S Bell, R Francis, D Beeby, J Finch
Aberdeen Royal Infirmary, Aberdeen	41	Prof M J Macleod PI, Dr G Guzman-Gutierrez, Dr K Carter, J Irvine
Royal United Hospital, Bath	40	Dr L Gbadamoshi PI, T Costa, S Heirons, H Stoney, L Shaw, J Choulerton, D Catibog
Sunderland Royal Hospital, Sunderland	39	Dr N Sattar PI, Dr M Myint, A Smith, K Serac
Royal Preston Hospital, Preston	38	Dr H Emsley PI, Dr C Anazodo, Dr S Sultan, B Gregary, A Brown
Maidstone Hospital, Maidstone	38	Dr A Mahmood PI, Dr N Chattha, Dr W Old, C Pegg, M Davey, M Page, B Sandhu, E Phiri
Yeovil District Hospital, Yeovil	37	Dr K Rashed PI, Dr E Wilson, Dr E Hindley, S Board, S Antony, A Tanate
Royal Victoria Infirmary, Newcastle	37	Dr M Davis PI, Dr A Dixit, V Slater, M Fawcett
Royal Derby Hospital, Derby	36	Dr T England PI, Dr J Scott, Dr J Beavan, A Hedstrom
Musgrove Park Hospital, Taunton	36	Dr D Karunatilake PI, K Gillmain, N Singh, T Hallows
University Hospital Monklands, Airdrie	36	Dr M Barber PI, Dr L Yates, Dr C Micallef, D Esson
Ninewells Hospital and Medical School, Dundee	34	Dr Wai Meng Yu PI, Dr B Jaa Ming New, Dr A Matos, C Burt, L Cabrelli, G Wilkie
Broomfield Hospital, Chelmsford	33	Dr M Meegada PI, Dr R Kirthivasan, C Fox, V Mead, A Lyle
Colchester General Hospital, Colchester	32	Dr R Saksena PI, A Bakshi, A O'Kelly
Kings Mill Hospital, Sutton-in-Ashfield	31	Dr J Rehan PI, Dr O Ebueka, Dr M Cooper, I Wynter, S Smith
Prince Philip Hospital, Llanelli	31	Dr S Kumar PI, L O'Brien, Cerrys Parker, Emma Parker
Bradford Royal Infirmary, Bradford	31	Dr N Khan PI, Dr C Patterson, Dr S Maguire, O Quinn, R Bellfield
Milton Keynes University Hospital, Milton Keynes	29	Dr Y Behnam PI, Dr J Costa, C Padilla-Harris, L Moram
Bronglais General Hospital, Aberystwyth	29	Dr S A Raza PI, H Tench, T Sims, H McGuinness, R Loosley, R Wolf-Roberts
Southmead Hospital, Bristol	29	Dr S Buddha PI, Dr I Salt, K Lewis
Whiston Hospital, Prescot	28	Dr S Mavinamne PI, C Ditchfield, S Dealing
Derriford Hospital, Plymouth	27	Dr A Shah PI, Dr G Crossingham, M Mwadeyi
University Hospital of Coventry, Coventry	27	Dr A Kenton PI, F Omoregie
Kent and Canterbury Hospital, Canterbury	27	Dr D Hargroves PI, Dr S Abubakar, A Warwick, G Hector
Leeds General Infirmary, Leeds	26	Dr S Maguire PI, Dr Hassan, E Veraque, M Farman, L Makawa
Forth Valley Royal Hospital, Larbert	26	Dr A Byrne PI, Dr J Kirkham, Dr G Blayney, Prof J Selwyn
Epsom General Hospital, Epsom	26	Dr P Kakar PI, Dr M Al Khaddour, R Dhami, E Baker
Hull Royal Infirmary, Hull	25	Dr B Esisi PI, E Clarkson, D Fellowes
Southend Hospital, Southend	24	Dr J Kresmir PI, Dr P Guyler, Dr D Ngo, Dr I Wijenayake, S Tysoe, J Galliford, P Harman
Northumbria Hexham, North Tyneside, Wansbeck, Cramlington	24	Dr M Garside PI, Dr M Badanahatti, A Smith, V Riddell
Gloucestershire Royal Hospital, Gloucester	24	Dr G Gramizadeh PI, Dr D Dutta, Dr M Bajoriene, Dr H Erdogan, D Ward

Royal Infirmary of Edinburgh, Edinburgh	20	Dr F Doubal PI, Dr N Samarasekera, S Risbridger, A MacRaild
West Suffolk Hospital, Bury-St-Edmunds	19	Dr A Azim PI, L Wood, R Tempest
Queen Elizabeth Hospital, King's Lynn	19	Dr R Shekhar PI, Dr U Rai, T Fuller, A Joshy, E Nadar
Calderdale and Huddersfield NHS Foundation Trust, Halifax	19	Dr M Kini PI, Dr S Ahmad, M Robinson, L King
Northampton General Hospital, Northampton	19	Dr V Srinivasan PI, Dr M Karwacka-Cichomska, V Moore, K Smith, B Kariyadil
Lincoln County Hospital, Lincoln	17	Dr K Kong PI, Dr K Jergovic, K Hubbard, S Arif
Peterborough City Hospital, Peterborough	17	Dr M Hasan PI, N Temple, D Arcoria, Z Horne
James Paget University Hospital, Great Yarmouth	16	Dr T Soe PI, Dr H Wyllie, C Hacon, H Sutherland
Arrowe Park Hospital, Birkenhead	15	Dr B Menezes PI, V Johnson
Royal Hampshire County Hospital, Winchester	14	Dr N Smyth PI, Dr Z Mehdi, Dr E Tone, A Bradley, E Levell
Great Western Hospital, Swindon	14	Dr A Ekkert PI, Dr S Mazzucco, L McCafferty, L Vonoven, S Dewan
Glangwili General Hospital, Carmarthen	13	Dr P Sridhar PI, J Thomas, S Coetzee, B Icke, J Williams
Fairfield General Hospital, Bury	13	Dr N Saravanan PI, P Bradley, R M Gibson, J Antony
Darent Valley Hospital, Dartford	13	Dr I Ashraf PI, J Mabutti, C Kamundi, P Patiola, N Oakley
Dorset County Hospital, Dorchester	12	Dr H Proeschel PI, Dr D Keely, W Longley, A Cave, C Ambrico
Salisbury District Hospital, Salisbury	11	Dr T Black PI, Dr E Porretta, A Anthony
Poole Hospital, Poole	11	Dr S Ragab PI, J Dube
Russell's Hall Hospital, Dudley	11	Dr S Kausar PI, Dr A Gujjar, D M Abdullah, D Kaur
Queen's Hospital, Romford	10	Dr N Gadapa PI, Dr S Choudhary, Dr N Nisar, G Fawehinmi, K Dunne, S King
Salford Royal Hospital, Salford	10	Dr A Kishore PI, S Lee, T Marsden, M Slaughter, K Cawley, J Perez
Doncaster Royal Infirmary, Doncaster	10	Dr P Anderton PI, Dr S Soussi, D Walstow, R Pugh
Royal Liverpool Hospital, Liverpool	9	Dr A Manoj PI, G Fletcher, P Lopez
Craigavon Area Hospital, Portadown	9	Dr M McCormick PI, Dr M Magee, Dr G Tallon, D McFarland, D Cosgrove
Norfolk and Norwich University Hospital, Norwich	9	Dr N Shinh PI, Dr K Metcalf, Dr A Kostyuk, S McDonald, S Sayers
Wrexham Maelor Hospital, Wrexham	8	Dr W Sayed PI, Dr S Abraham, G Szabo, G Crosbie
Royal Victoria Hospital, Belfast	6	Dr J McIlmoyle PI, Dr P Fearon, K Courtney, S Tauro
Royal Blackburn Hospital, Blackburn	6	Dr A Singh PI, Dr A Nair, S Duberley, S Philip, C Curley, W Goddard
York General Hospital, York	5	Dr Luke Bridge PI, Dr P Wilcoxson, Dr P Wanklyn, J Owen
Torbay Hospital, Torquay	5	Dr J France PI, B Reed, A Foulds
Nevill Hall Hospital, Abergavenny	5	Dr B Richard PI, L Parfitt
St. Peter's Hospital, Chertsey	4	Dr B Affley PI, Dr C Russo, M Dsouza, E Cruddas
William Harvey Hospital, Ashford	3	Dr D Hargroves PI, J Rand

Royal Gwent Hospital - The Grange, Newport	3	Dr S Shekar PI, Dr Y Bhat, G Marshall, M Nash
New Cross Hospital, Wolverhampton	3	Dr N Ahmad PI, B O Okoko, R Evans, T Taylor
Queen Elizabeth University Hospital, Glasgow	2	Dr J Dawson PI, E Colquhoun
Withybush General Hospital, Haverford West	1	Dr C James PI, Dr C Aguirre, C MacPhee, J Phipps
Sandwell General Hospital, West Bromwich	1	Dr S Ispoglou PI, A Hayes, R Evans

Table S2: Characteristics of included and excluded participants

	Included (n=3572)	Excluded (n=76)
Age	78·0 (9·9)	77·9 (9·9)
Sex	1620 (45·4%)	32 (42·1%)
Ethnicity		
White	3347 (93·7%)	70 (92·1%)
Black British; African or Caribbean	58 (1·6%)	0 (0%)
South Asian	60 (1·7%)	0 (0%)
East Asian or Southeast Asian	39 (1·1%)	2 (2·6%)
Mixed ethnicity; other; not disclosed or missing	68 (1·9%)	4 (5·3%)
Hypertension	2402 (67·2%)	53 (69·7%)
Diabetes type 1 or 2; known prior to stroke or diagnosed during admission	751 (21·0%)	24 (31·6%)
Hypercholesterolemia	1171 (32·8%)	28 (36·8%)
Known chronic kidney disease	534 (14·9%)	13 (17·1%)
Type of Atrial fibrillation		
Paroxysmal	955 (26·7%)	19 (25·0%)
Persistent	2525 (70·7%)	52 (68·4%)
Atrial Flutter	91 (2·5%)	2 (2·6%)
Age of AF		
Newly diagnosed	1758 (49·2%)	33 (45·2%)
Known prior to stroke	1814 (50·8%)	40 (54·8%)
Myocardial infarction	329 (9·2%)	11 (14·5%)
History of angina	257 (7·2%)	7 (9·2%)
Coronary revascularisation	222 (6·2%)	11 (14·5%)
Congestive heart failure	375 (10·5%)	9 (11·8%)
Peripheral arterial disease	77 (2·2%)	2 (2·6%)
Previous ischaemic stroke	532 (14·9%)	10 (13·2%)
Previous other intracranial bleeding	63 (1·8%)	1 (1·3%)
Known dementia/cognitive impairment	240 (6·7%)	9 (11·8%)
Current or former smoker	1279 (37·3%)	25 (37·9%)
Current alcohol intake >14 units per week	398 (11·1%)	6 (7·9%)
Previous anticoagulation	1263 (35·4%)	30 (39·5%)
Vitamin K antagonist	110 (8·7%)	5 (16·7%)
Direct oral anticoagulant	1153 (91·3%)	25 (83·3%)
Previous antiplatelet use	400 (11·2%)	10 (13·2%)
IV Thrombolysis	786 (22·0%)	15 (19·7%)
Endovascular treatment	263 (7·4%)	3 (3·9%)
NIHSS Score upon admission	5 (3 to 10)	6 (3 to 12)
NIHSS score at randomisation	4 (2 to 7)	4 (2 to 9)
Blood pressure (Sys)	134·3 (19·2)	138·6 (20·4)
Blood pressure (Dia)	76·4 (12·8)	77·1 (13·0)
Pre-Stroke Modified Rankin Score (mRS)	0 (0 to 2)	1 (0 to 2)
Timing of DOAC initiation		
Delayed	1780 (49·8%)	44 (57·9%)
Early	1792 (50·2%)	32 (42·1%)

Table S3: Baseline characteristics according to infarct volume categories

Infarct volume category	Not visible (n=911)	<5ml (n=1174)	5-10ml (n=443)	10-25ml (n=570)	25-50ml (n=289)	>50ml (n=185)
Age	80.2 (9.1)	77.9 (9.4)	76.6 (11.2)	76.7 (10.1)	77.0 (9.5)	75.8 (11.4)
Sex; female	441 (48.4%)	530 (45.1%)	220 (49.7%)	245 (43.0%)	114 (39.4%)	70 (37.8%)
Ethnicity						
White	870 (95.5%)	1093 (93.1%)	412 (93.0%)	527 (92.5%)	274 (94.8%)	171 (92.4%)
Black	9 (1.0%)	20 (1.7%)	9 (2.0%)	10 (1.8%)	6 (2.1%)	4 (2.2%)
South Asian	7 (0.8%)	25 (2.1%)	5 (1.1%)	15 (2.6%)	5 (1.7%)	3 (1.6%)
East/Southeast Asian	8 (0.9%)	15 (1.3%)	6 (1.4%)	6 (1.1%)	0 (0%)	4 (2.2%)
Mixed/other/undisclosed	17 (1.9%)	21 (1.8%)	11 (2.5%)	12 (2.1%)	4 (1.4%)	3 (1.6%)
Hypertension	631 (69.3%)	802 (68.3%)	302 (68.2%)	367 (64.4%)	190 (65.7%)	110 (59.5%)
Diabetes	196 (21.5%)	256 (21.8%)	80 (18.1%)	116 (20.4%)	61 (21.1%)	42 (22.7%)
Hypercholesterolaemia	303 (33.3%)	397 (33.8%)	131 (29.6%)	182 (31.9%)	99 (34.3%)	59 (31.9%)
Chronic kidney disease	152 (16.7%)	180 (15.3%)	59 (13.3%)	76 (13.3%)	40 (13.8%)	27 (14.6%)
AF type						
Paroxysmal	254 (27.9%)	337 (28.7%)	104 (23.5%)	143 (25.1%)	66 (22.8%)	51 (27.6%)
Persistent	632 (69.4%)	803 (68.4%)	331 (74.7%)	411 (72.1%)	216 (74.7%)	132 (71.4%)
Atrial Flutter	25 (2.7%)	33 (2.8%)	8 (1.8%)	16 (2.8%)	7 (2.4%)	2 (1.1%)
AF diagnosis						
Newly diagnosed	377 (41.4%)	584 (49.7%)	226 (51.0%)	310 (54.4%)	158 (54.7%)	103 (55.7%)
Known prior to stroke	534 (58.6%)	590 (50.3%)	217 (49.0%)	260 (45.6%)	131 (45.3%)	82 (44.3%)
Myocardial infarction	104 (11.4%)	95 (8.1%)	42 (9.5%)	46 (8.1%)	28 (9.7%)	14 (7.6%)
Angina	83 (9.1%)	92 (7.8%)	19 (4.3%)	34 (6.0%)	12 (4.2%)	17 (9.2%)
Coronary revascularisation	64 (7.0%)	69 (5.9%)	22 (5.0%)	36 (6.3%)	19 (6.6%)	12 (6.5%)
Congestive HF	117 (12.8%)	116 (9.9%)	44 (9.9%)	53 (9.3%)	24 (8.3%)	21 (11.4%)
Peripheral arterial disease	23 (2.5%)	21 (1.8%)	9 (2.0%)	13 (2.3%)	6 (2.1%)	5 (2.7%)
Previous IS	169 (18.6%)	180 (15.3%)	58 (13.1%)	62 (10.9%)	36 (12.5%)	27 (14.6%)
Previous ICH	20 (2.2%)	21 (1.8%)	9 (2.0%)	7 (1.2%)	5 (1.7%)	1 (0.5%)
Known cognitive impairment	92 (10.1%)	65 (5.5%)	30 (6.8%)	29 (5.1%)	17 (5.9%)	7 (3.8%)
Current/former smoker	318 (36.5%)	426 (37.4%)	148 (34.9%)	214 (39.3%)	105 (37.4%)	68 (39.1%)
Alcohol intake > 14 units/week	92 (10.1%)	134 (11.4%)	56 (12.6%)	54 (9.5%)	37 (12.8%)	25 (13.5%)
Previous anticoagulation	391 (42.9%)	427 (36.4%)	143 (32.3%)	157 (27.5%)	90 (31.1%)	55 (29.7%)
VKA	35 (9.0%)	35 (8.2%)	11 (7.7%)	16 (10.2%)	8 (8.9%)	5 (9.1%)
DOAC	356 (91.0%)	392 (91.8%)	132 (92.3%)	141 (89.8%)	82 (91.1%)	50 (90.9%)
Previous antiplatelet	109 (12.0%)	130 (11.1%)	44 (9.9%)	62 (10.9%)	34 (11.8%)	21 (11.4%)
IV thrombolysis	213 (23.4%)	209 (17.8%)	109 (24.6%)	131 (23.0%)	70 (24.2%)	54 (29.2%)

Endovascular treatment	53 (5.8%)	72 (6.1%)	42 (9.5%)	50 (8.8%)	23 (8.0%)	23 (12.4%)
DOAC initiation						
Delayed	430 (47.2%)	603 (51.4%)	225 (50.8%)	285 (50.0%)	145 (50.2%)	92 (49.7%)
Early	481 (52.8%)	571 (48.6%)	218 (49.2%)	285 (50.0%)	144 (49.8%)	93 (50.3%)
NIHSS on admission, median (IQR)	5.0 (3.0 to 9.0)	4.0 (2.0 to 8.0)	6.0 (3.0 to 11.0)	6.0 (3.0 to 12.0)	7.0 (4.0 to 13.0)	13.0 (7.0 to 19.0)
NIHSS on randomisation						
0-4	532 (58.4%)	809 (68.9%)	259 (58.5%)	308 (54.0%)	125 (43.3%)	35 (18.9%)
5-10	264 (29.0%)	280 (23.9%)	125 (28.2%)	164 (28.8%)	104 (36.0%)	56 (30.3%)
11-15	64 (7.0%)	48 (4.1%)	41 (9.3%)	54 (9.5%)	35 (12.1%)	33 (17.8%)
16-21	35 (3.8%)	33 (2.8%)	12 (2.7%)	35 (6.1%)	19 (6.6%)	39 (21.1%)
>21	16 (1.8%)	4 (0.3%)	6 (1.4%)	9 (1.6%)	6 (2.1%)	22 (11.9%)
Median (IQR)	4.0 (2.0 to 7.0)	3.0 (1.0 to 5.0)	4.0 (2.0 to 8.0)	4.0 (2.0 to 8.0)	5.0 (3.0 to 10.0)	11.0 (6.0 to 18.0)
sBP, mmHg, mean (SD)	134.9 (19.1)	134.5 (19.4)	132.7 (19.0)	133.8 (18.7)	134.9 (20.5)	134.7 (19.2)
dBP, mmHg, mean (SD)	75.9 (12.0)	76.4 (13.0)	77.3 (12.9)	76.7 (13.1)	77.1 (13.0)	75.9 (13.7)
Pre-stroke mRS, median (IQR)	1 (0-2)	0 (0-2)	0 (0-2)	0 (0-1)	0 (0-1)	0 (0-2)
Infarct volume, ml, median (IQR)	0.0 (0.0 to 0.0)	1.5 (0.6 to 2.9)	7.1 (6.1 to 8.3)	15.9 (12.7 to 19.7)	32.4 (28.7 to 39.3)	77.4 (61.2 to 98.8)
Infarct volume determined using MRI	36 (4.0%)	631 (53.8%)	172 (38.8%)	173 (30.3%)	70 (24.2%)	17 (9.2%)

Table S4: Secondary outcomes according to infarct characteristics

Recurrent ischaemic stroke	Events / participants<br>Early DOAC (n = 1792)	 Delayed DOAC (n = 1780)	Odds ratio (95% CI)	$p_{interaction}$
Total events	42	41		
Infarct volume category				0·214
No visible infarct	11/481 (2·3%)	9/430 (2·8%)	1·09 (0·45-2·67)	
Visible infarct < 5 ml	14/568 (2·5%)	8/599 (2·1%)	1·87 (0·78-4·49)	
5-10 ml	3/220 (1·4%)	9/229 (3·9%)	0·34 (0·09-1·26)	
10-25 ml	10/285 (3·5%)	5/283 (1·8%)	2·02 (0·68-5·98)	
25-50 ml	4/145 (2·8%)	5/145 (3·4%)	0·80 (0·21-3·04)	
>50 ml	0/93 (0%)	5/94 (5·3%)	Inestimable	
ELAN classification				0·391
Minor stroke	18/732 (2·5%)	13/709 (1·8%)	1·35 (0·66-2·78)	
Moderate stroke	15/814 (1·8%)	21/823 (2·6%)	0·72 (0·37-1·40)	
Major stroke	9/246 (3·7%)	7/248 (2·8%)	1·30 (0·48-3·56)	
Arterial territory				0·181
Anterior circulation	18/997 (1·8%)	25/1033 (2·4%)	0·75 (0·40-1·38)	
Posterior circulation	8/218 (3·7%)	6/221 (2·7%)	1·35 (0·46-3·95)	
Multi-territory	5/96 (5·2%)	1/96 (1·0%)	5·33 (0·61-46·6)	
**Symptomatic ICH**				
Total events	11	12		
Infarct volume category				0·936
No visible infarct	1/481 (0·2%)	0/430 (0%)	Inestimable	
Visible infarct < 5 ml	2/568 (0·4%)	2/599 (0·3%)	1·04 (0·15-7·46)	
5-10 ml	2/220 (0·9%)	1/229 (0·4%)	1·97 (0·17-22·2)	
10-25 ml	3/285 (1·1%)	4/283 (1·4%)	0·73 (0·16-3·36)	
25-50 ml	2/145 (1·4%)	3/145 (2·1%)	0·63 (0·10-3·93)	
>50 ml	1/93 (1·1%)	2/94 (2·1%)	Inestimable	
ELAN classification				0·544
Minor stroke	1/732 (0·1%)	0/709 (0%)	Inestimable	
Moderate stroke	7/814 (0·9%)	7/823 (0·9%)	0·99 (0·34-2·85)	
Major stroke	3/246 (1·2%)	5/248 (2·0%)	Inestimable	
Arterial territory				0·959
Anterior circulation	8/997 (0·8%)	8/1033 (0·8%)	1·00 (0·37-2·74)	
Posterior circulation	2/218 (0·9%)	2/221 (0·9%)	1·06 (0·14-7·85)	
Multi-territory	0/96 (0%)	2/96 (2·1%)	Inestimable	

Table S5: DOAC initiation timing according to treatment allocation and subgroups

	Early DOAC		Delayed DOAC	
**Subgroups**	**n**	**Days to DOAC initiation, median (IQR)**	**n**	**Days to DOAC initiation, median (IQR)**
Infarct volume categories				
No visible infarct	481	3 (2 to 4)	430	8 (7 to 9)
Visible infarct < 5 ml	568	3 (3 to 4)	599	8 (7 to 9)
5-10 ml	220	3 (3 to 4)	229	8 (7 to 9)
10-25 ml	285	3 (3 to 4)	283	8 (7 to 10)
25-50 ml	145	3 (3 to 4)	145	8 (7 to 10)
>50 ml	93	3 (3 to 4)	94	9 (8 to 12)
ELAN classification				
Minor stroke	731	3 (3 to 4)	709	8 (7 to 9)
Moderate stroke	815	3 (3 to 4)	823	8 (7 to 9)
Major stroke	246	3 (3 to 4)	248	8 (7 to 11)
Arterial territory				
Anterior circulation	998	3 (3 to 4)	1033	8 (7 to 9)
Posterior circulation	218	3 (3 to 4)	221	8 (7 to 9)
Multi-territory	95	3 (3 to 4)	96	8 (7 to 10)
Unknown	481	3 (3 to 4)	430	8 (7 to 9)

Figure S1: Representative case examples of infarct segmentations from different volume categories used in the analysis

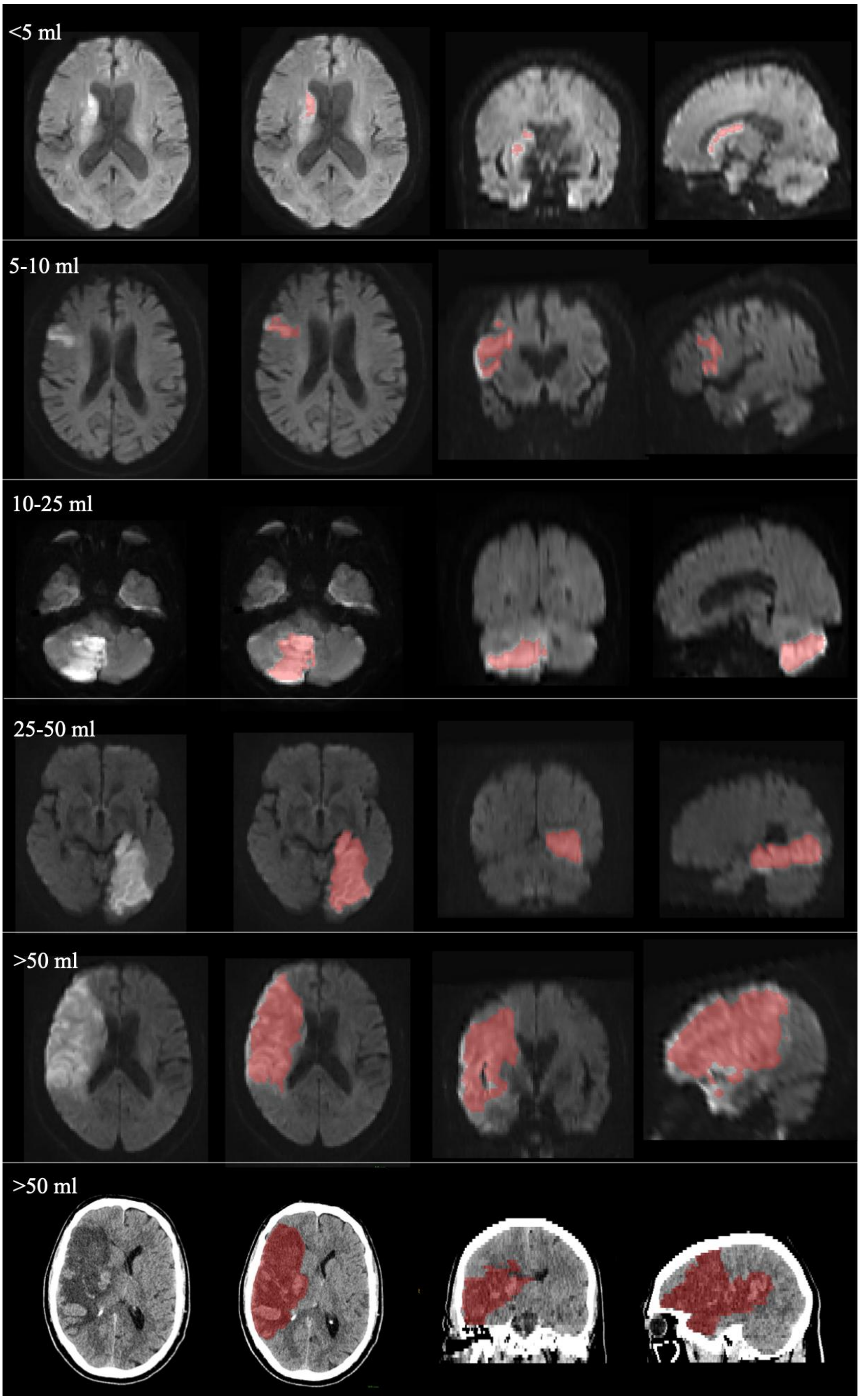

Infarct volumes in order from top to bottom: 2.8, 6.9, 22.7, 40.8, 158.0 and 253.5 ml (with parenchymal haematoma grade 1 haemorrhagic transformation on the Heidelberg scale)

Figure S2: Treatment effect of early versus delayed anticoagulation with respect to the secondary outcome of recurrent ischaemic stroke

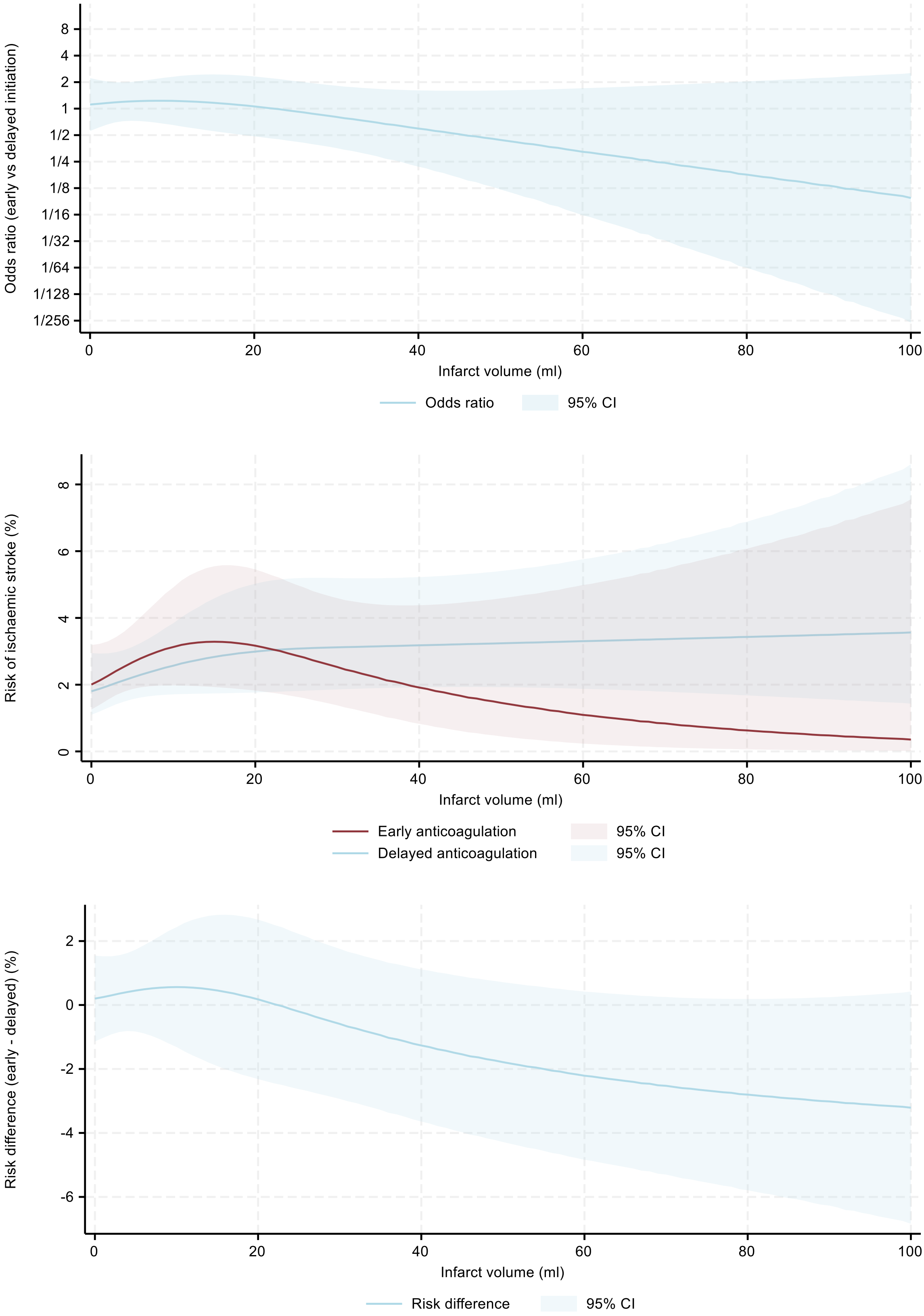

For display purposes, the x-axis is truncated at 100mls. Only 46 participants had an infarct volume >100mls; there were no recurrent ischaemic strokes in those participants.

Figure S3: Treatment effect of early versus delayed anticoagulation with respect to the secondary outcome of symptomatic intracranial haemorrhage

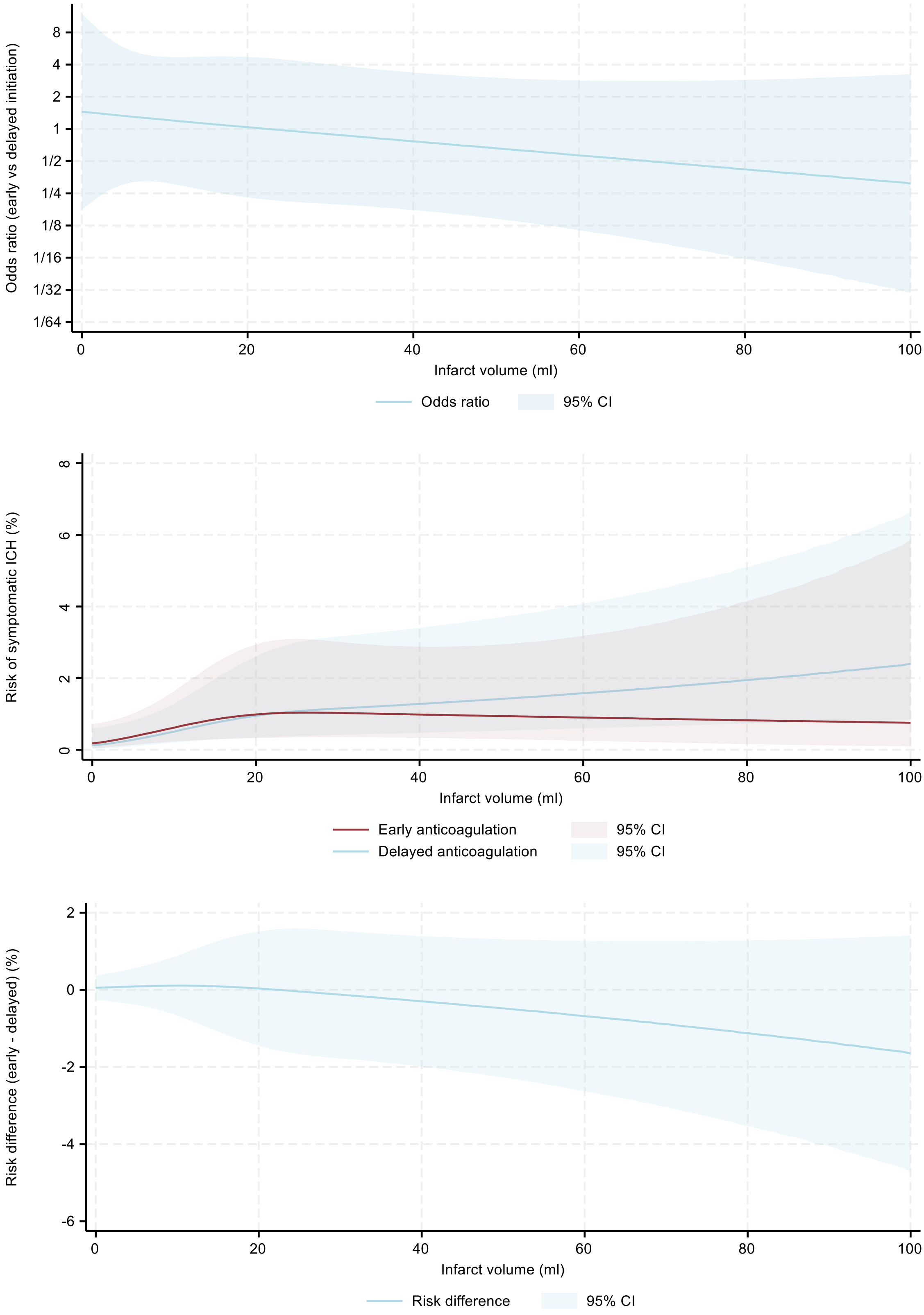

For display purposes, the x-axis is truncated at 100mls. Only 46 participants had an infarct volume >100mls; one symptomatic ICH occurred in those participants.

Figure S4: Heatmaps showing voxel-based density of infarcted tissue according to treatment allocation

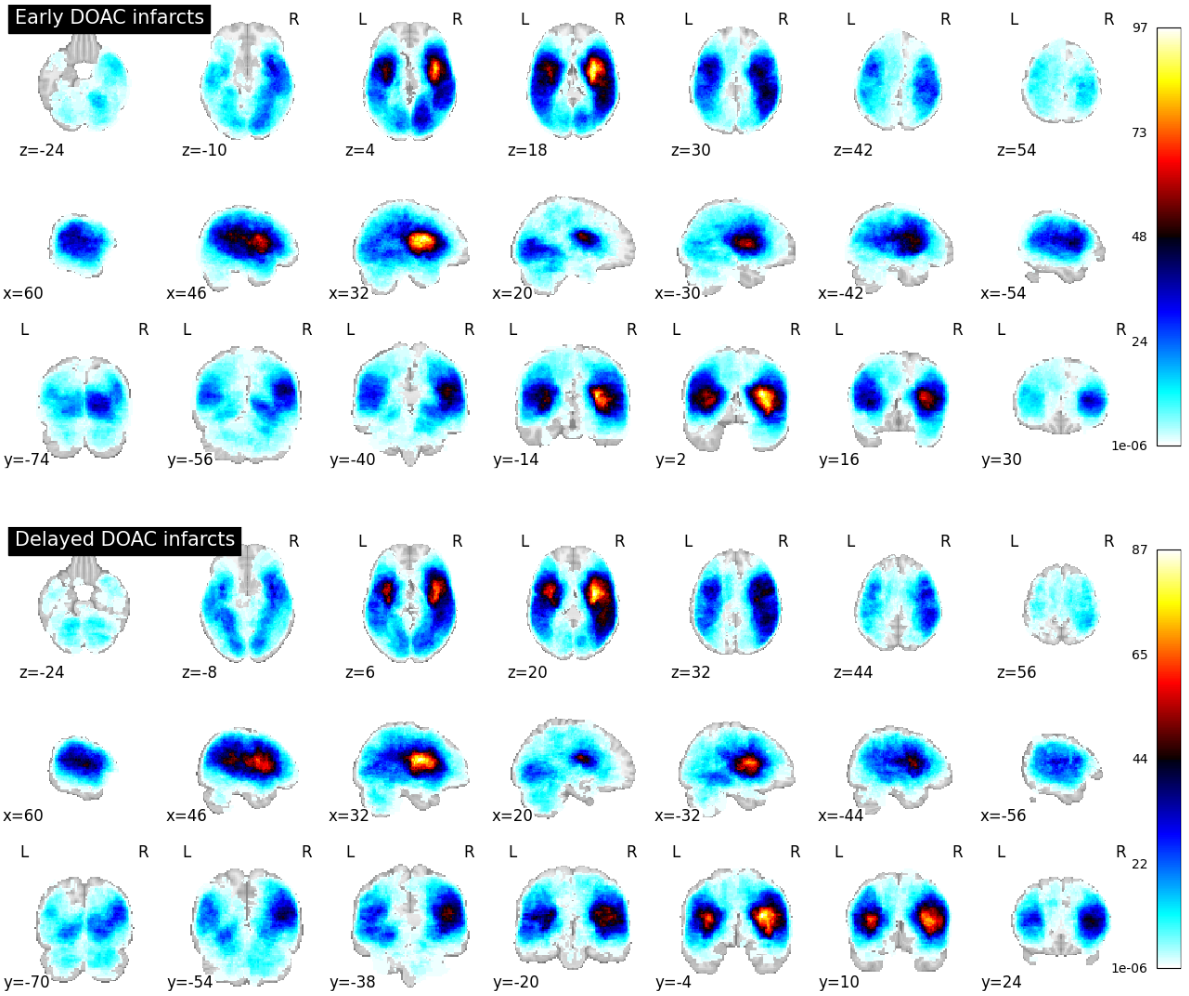

Figure S5: Sensitivity analysis showing treatment effect of early versus delayed anticoagulation with respect to the primary outcome according to infarct volume, omitting participants with only a CT within six hours of stroke onset

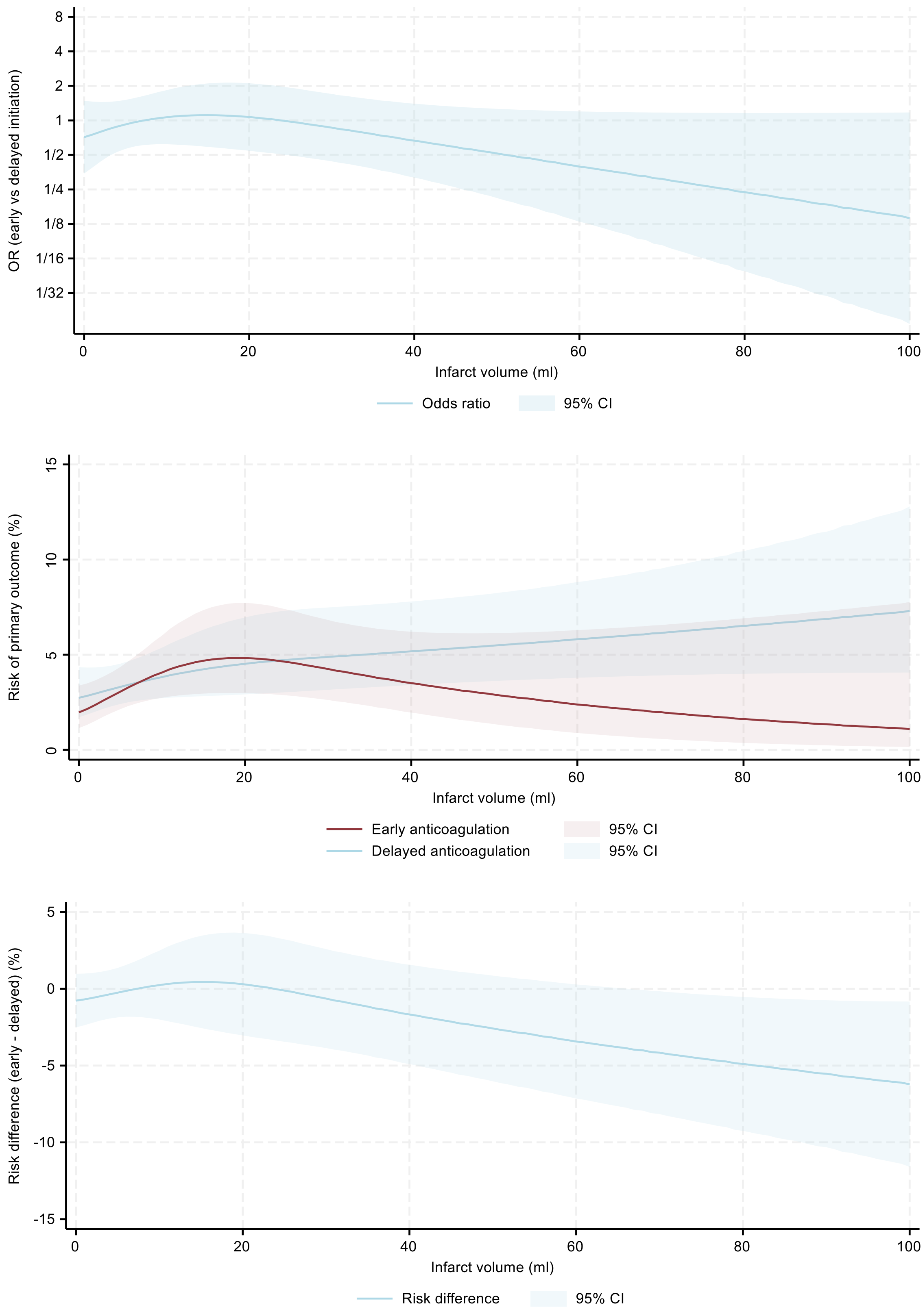

Supplementary References

1. Brudfors M, Balbastre Y, Nachev P, Ashburner J. A Tool for Super-Resolving Multimodal Clinical MRI [Internet]. arXiv; 2019 [cited 2025 Jun 12]. Available from: http://arxiv.org/abs/1909.01140

2. Brudfors M, Balbastre Y, Nachev P, Ashburner J. MRI Super-Resolution Using Multi-channel Total Variation. In: Nixon M, Mahmoodi S, Zwiggelaar R, editors. Medical Image Understanding and Analysis. Cham: Springer International Publishing; 2018. p. 217–28
