# Supplementary figures and images for: Early versus delayed anticoagulation in acute ischemic stroke with atrial fibrillation according to infarct volume and location: A prespecified subgroup analysis of the OPTIMAS randomized controlled trial
Source: Int J Stroke. 2026 Mar 30;21(7):956–67. doi: 10.1177/17474930261441297 (PMC13392158; doi:10.1177/17474930261441297)

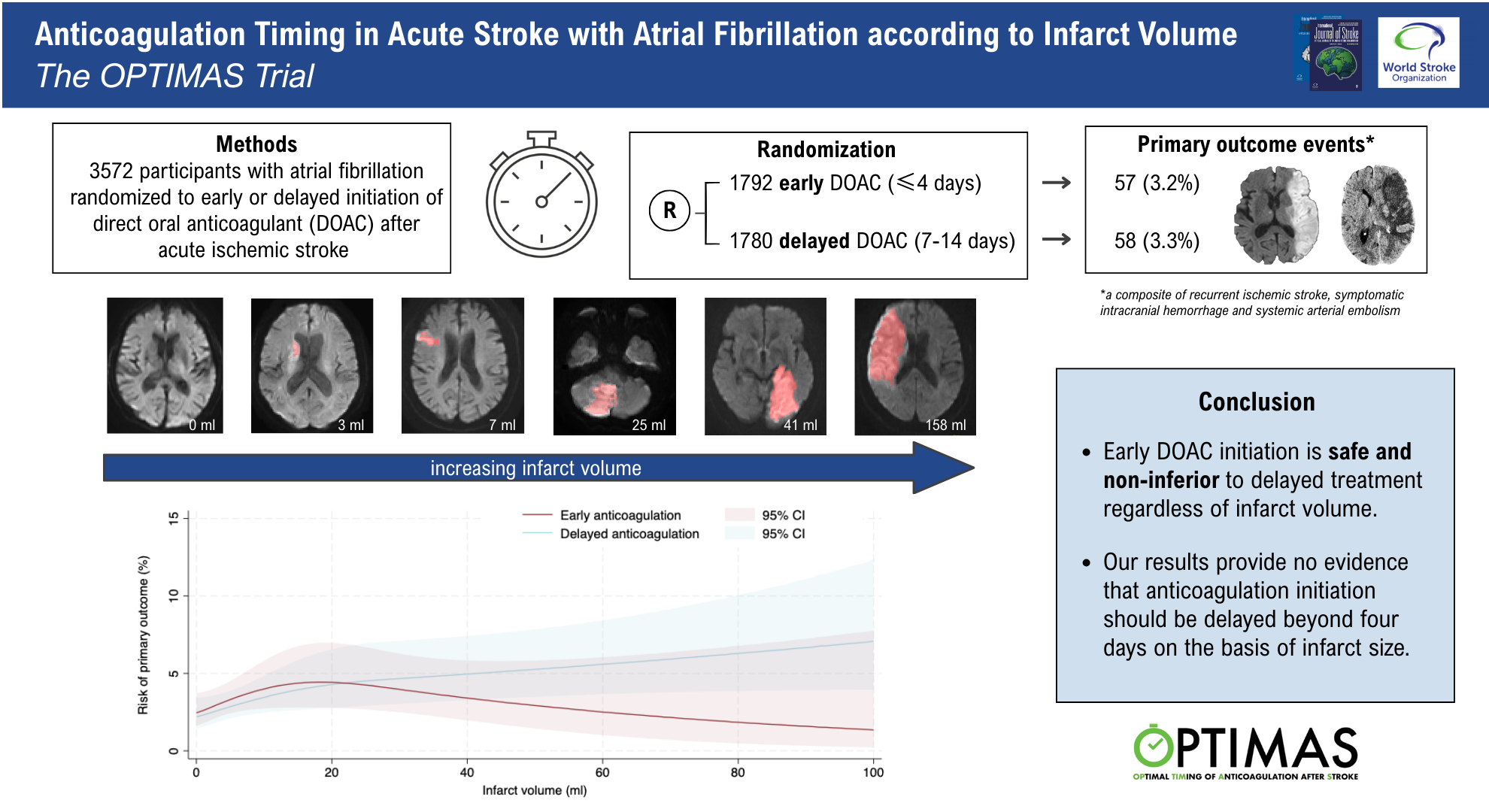

Supplement: sj-png-2-wso-10.1177_17474930261441297 – Supplemental material for Early versus delayed anticoagulation in acute ischemic stroke with atrial fibrillation according to infarct volume and location: A prespecified subgroup analysis of the OPTIMAS randomized controlled trial [file sj-png-2-wso-10.1177_17474930261441297.png]
